# Pupillary reflex measurement predicts insufficient analgesia before endotracheal suctioning in critically ill patients

**DOI:** 10.1186/cc12840

**Published:** 2013-07-24

**Authors:** Jerome Paulus, Antoine Roquilly, Hélène Beloeil, Julien Théraud, Karim Asehnoune, Corinne Lejus

**Affiliations:** 1 Centre Hospitalier Universitaire de Nantes, Service d’Anesthésie Réanimation Chirurgicale, Hôtel Dieu Hôpital Mère Enfant, 1 Place Alexis Ricordeau, Nantes F-44093, France; 2 Anesthesiology and Surgical Critical Care Medicine, Centre Hospitalier Universitaire de Nantes, Service d’Anesthésie Réanimation Chirurgicale, Hôtel Dieu Hôpital Mère Enfant, 1 Place Alexis Ricordeau, Nantes, F-44093, France; 3 Centre Hospitalier Universitaire de Rennes, Service d’Anesthésie et de Réanimation, Inserm UMR 991, 2 Rue Henri le Guilloux, Rennes, F-35033, France; 4 Centre Hospitalier Universitaire de Nantes, Service d’Anesthésie Réanimation Chirurgicale, Hôtel Dieu Hôpital Mère Enfant, 1 Place Alexis Ricordeau, Nantes, France

## Abstract

**Introduction:**

This study aimed to evaluate the pupillary dilatation reflex (PDR) during a tetanic stimulation to predict insufficient analgesia before nociceptive stimulation in the intensive care unit (ICU).

**Methods:**

In this prospective non-interventional study in a surgical ICU of a university hospital, PDR was assessed during tetanic stimulation (of 10, 20 or 40 mA) immediately before 40 endotracheal suctionings in 34 deeply sedated patients. An insufficient analgesia during endotracheal suction was defined by an increase of ≥1 point on the Behavioral Pain Scale (BPS).

**Results:**

A total of 27 (68%) patients had insufficient analgesia. PDR with 10 mA, 20 mA and 40 mA stimulation was higher in patients with insufficient analgesia (*P* <0.01). The threshold values of the pupil diameter variation during a 10, 20 and 40 mA tetanic stimulation to predict insufficient analgesia during an endotracheal suctioning were 1, 5 and 13% respectively. The areas (95% confidence interval) under the receiver operating curve were 0.70 (0.54 to 0.85), 0.78 (0.61 to 0.91) and 0.85 (0.721 to 0.954) with 10, 20 and 40 mA tetanic stimulations respectively. A sensitivity analysis using the Richmond Agitation Sedation Scale (RASS) confirmed the results. The 40 mA stimulation was poorly tolerated.

**Conclusions:**

In deeply sedated mechanically ventilated patients, a pupil diameter variation ≥5% during a 20 mA tetanic stimulation was highly predictable of insufficient analgesia during endotracheal suction. A 40 mA tetanic stimulation is painful and should not be used.

## Introduction

A majority of patients in the intensive care unit (ICU) experiences endotracheal tube discomfort [1]. In the ICU setting, procedural procedures like endotracheal suction are frequent causes of acute pain [1-3]. Acute pain induces prolonged stress on biologic systems and may alter the outcome and the quality of life even after the patient’s discharge [4]. Predicting insufficient analgesia before a painful stimulation in deeply sedated patients in the ICU is challenging [5]. Indeed, in the Dolorea study, only 42% of patients received pain assessments on day 2 in ICUs, although 90% of patients are concomitantly given opioids [6]. However, pain assessment is associated with a reduction of the time on a ventilator and of the length of stay in the ICU [7]. New pain assessment tools have been recently described, but no one enables the adaption of analgesic infusion before a nociceptive stimulation.

The automatic video pupillometer is based on the pupillary dilatation reflex (PDR). A noxious stimulation dilates the pupils in anesthetized and awake patients. Larson *et al*. [8] first reported that the PDR allows assessment of the reaction to a painful stimulus during general anesthesia. Constant *et al*. [9] confirm that PDR is an earlier and more sensitive response predictor of analgesia than the hemodynamic changes or the bispectral index (BIS) in children under general anesthesia. In the immediate postoperative period, the PDR is significantly correlated with the verbal rating scale [10]. The authors concluded that the PDR could be useful to assess pain in patients with whom direct communication is difficult. Predicting insufficient analgesia before a nociceptive stimulation would be useful in the ICU especially before procedural pain, and the measure of the PDR with video pupillometry coupled with a tetanic stimulation has not been evaluated for that purpose in the ICU. The tetanic stimulation is a calibrated stimulation that may dilate the pupil and enable the assessment of the PDR, without excessive pain stimulation. We therefore aimed to assess the predictive value of the PDR during a tetanic stimulation as an indicator of insufficient analgesia before performing an endotracheal suction.

## Materials and methods

After ethical committee approval (Groupe Nantais d’Ethique dans le Domaine de la Santé, number 2011-07-02), a next of kin written informed consent was obtained. Retrospective consent, when available, was obtained from patients. This prospective study was conducted in a surgical ICU in a teaching hospital in Nantes (France). Patients aged between 18 and 85 years, under mechanical ventilation, with a pain level ≥3 on the Behavioral Pain Scale (BPS, a tool assessing pain in sedated patients) [11], and ≤−4 on the Richmond Agitation Sedation Scale (RASS) [12] were included. Several scales, including the BPS are used in the ICU [13]. The BPS is a behavior scale validated and reliable for critically ill patients [14]. Non-inclusion criteria were as follows: (1) refusal of the patient’s relatives, (2) hemodynamic instability, (3) previous pupil abnormality, (4) treatment with beta-blockers and (5) treatment that could interfere with the PDR.

**Figure 1:**
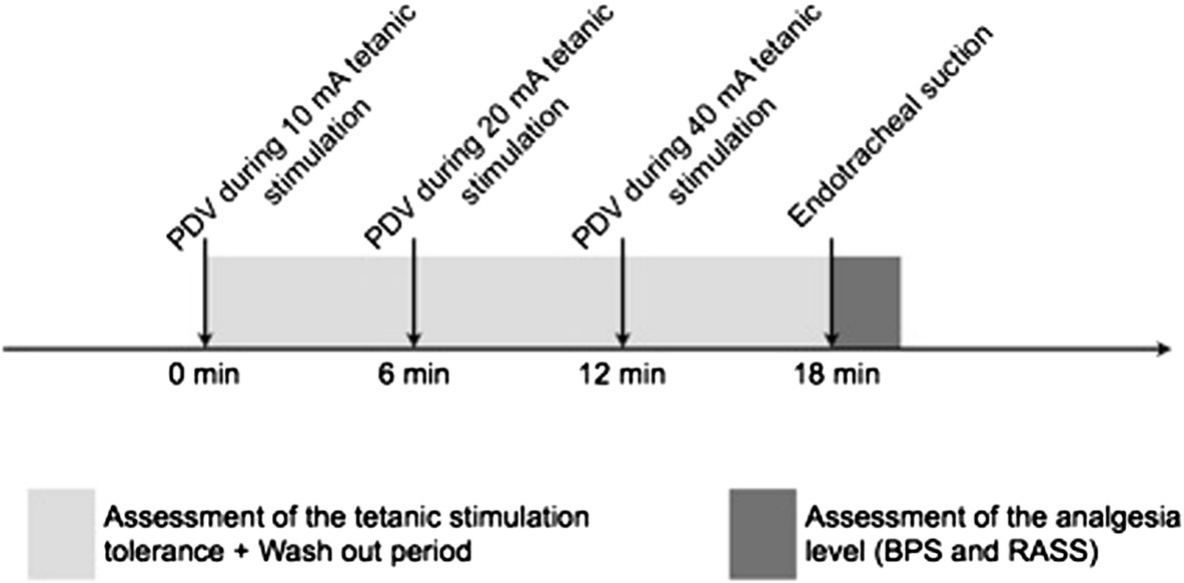
**Study design.** BPS, Behavioral Pain Scale; PDV, pupil diameter variation; RASS, Richmond Agitation Sedation Scale.

Pupil diameter variation was measured with a portable infrared pupillometer™ (IDMED, Marseille, France). This device performs multiple scanning of pupil diameter, displaying the percentage of pupil diameter variation within 10 seconds of tetanic stimulation of the median nerve. The median nerve provides feeling to the skin of the hand, including the middle finger, half of the ring finger, as well as the thumb and index finger. Ranges for stimulation intensity are 0 to 40 milliamps (mA). The system impedance level was controlled before the stimulation. The lighting in the room was set to avoid light shining directly into the patient’s eyes. The attending physician determined the depth of sedation using the RASS and BPS, cardiac and respiratory rates and systolic arterial pressure. The variation of the pupil diameter was then measured during a tetanic stimulation of 10 mA. As the tetanic stimulation could be painful by itself, the BPS, RASS and physiological values were recorded in the subsequent 60 seconds. The BPS and RASS were blinded to the PRD results. A 5-minute washout period (when hemodynamic values returned to normal) was respected before repeating this procedure with a 20 mA and a 40 mA tetanic stimulation and finally during an endotracheal suction (see Figure 1).

### Statistical analysis

Analysis was completed with SAS 9.1 statistical software (SAS Institute, Cary, NC, USA). The endpoint was the proportion of patients with an insufficient analgesia during an endotracheal suction, defined as an increase ≥1 point on the BPS. To confirm the robustness of the results, a sensitivity analysis was performed by using the RASS. An increase ≥1 point was also considered as insufficient analgesia. We aimed to determine the best value of pupil variation diameter during a 10, 20 or 40 mA tetanic stimulation that could predict an insufficient analgesia. A receiver operating curve was constructed by plotting sensitivity against the false positive (1 – specificity) over a range of cut-point values of the pupil diameter variation. The threshold associated with the best relationship between sensitivity and specificity was defined with the Youden index. The Youden index (sensitivity + specificity – 1) evaluates the performance of the PRD in predicting insufficient analgesia before endotracheal suctioning. Numerical variables were reported as medians (25^th^ to 75^th^ percentile) or mean (standard deviation, SD) and categorical variables as an absolute number (percentage). Chi-square tests, Fisher tests and Wilcoxon rank sum test were used as appropriate. A two-tailed *P* value <0.05 was considered significant.

## Results

### Study population

**Figure 2:**
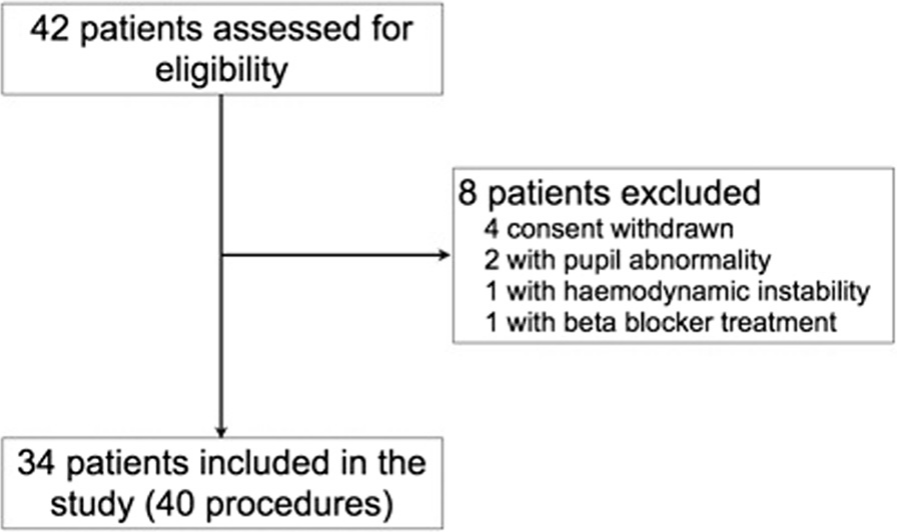
Flow diagram of included patients.

Forty measures were performed on 34 consecutive patients (see Figure 2). The demographic characteristics are provided in Table 1. Midazolam was used in 19 (56%) patients with a mean dose of 0.33 (0.10) mg/kg/h and propofol in 15 (44%) patients with a mean dose of 5 (1) mg/kg/h. Fentanyl was the opioid used in the whole population with a mean dose of 3 (1) μg/kg/h.

**Table 1:** General characteristics (n= 34)

	**Value**
Age (yr), mean (SD)	56 (19)
Sex ratio male/female, n (%)	22/12 (64.7/35.3)
Body mass index (kg/m^2^), mean (SD),	24.7 (4.4)
Simplified Acute Physiology Score II mean (SD)	29 (13)
Sequential Organ Failure Assessment score, median (25-75^th^ percentile)	3 (1-4)
McCabe Scale, n (%)	
A	26 (76.5)
B	7 (20.6)
C	1 (2.9)
Reasons for hospitalization, n (%)	
Postoperative	17 (50)
Traumatic brain injury	6 (17.7)
Sepsis	5 (14.7)
Multiple trauma	5 (14.7)
Stroke	1 (2.9)
Sedatives/opioids during the procedure, n (%):	
Propofol/fentanyl	15 (44)
Midazolam/fentanyl	19 (56)

*n* number of patients, *%* percentage of patients. *SD* standard deviation.

### Pupil diameter variation is different in patients with adequate or insufficient analgesia

**Figure 3:**
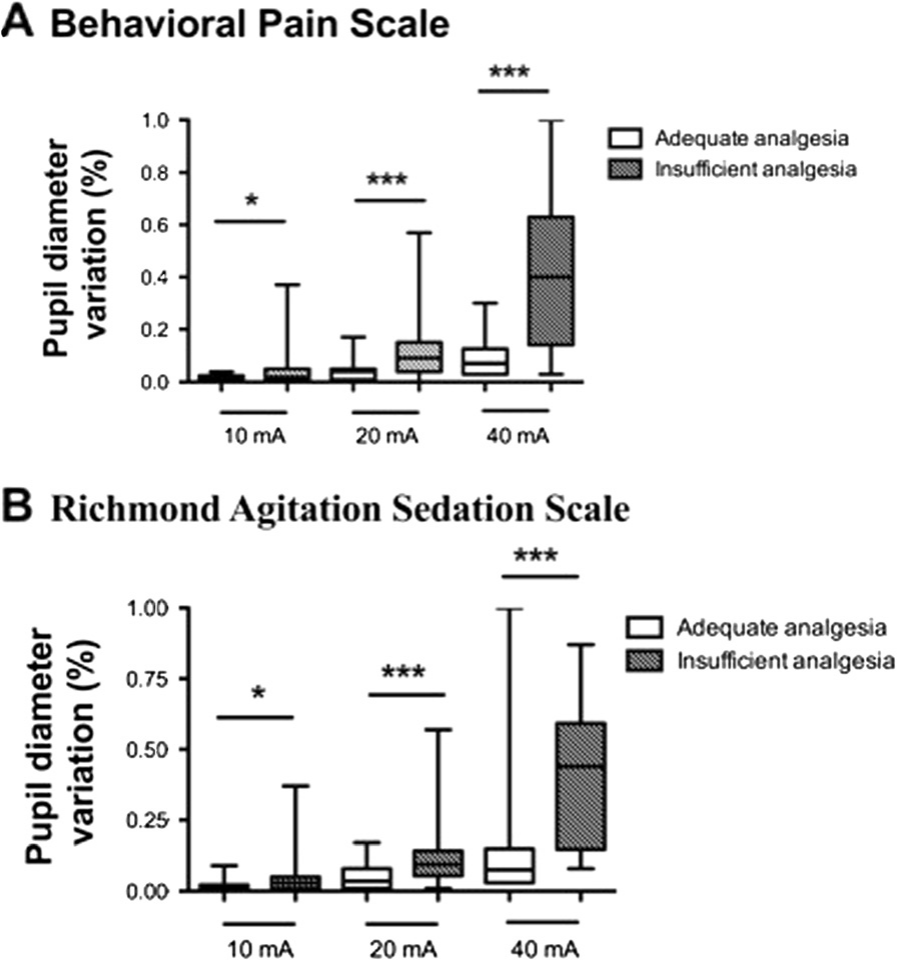
**Comparison of the pupil diameter variation assessed after a 10, 20 or 40 mA tetanic stimulation during 40 measurements in 34 critically ill patients with adequate or insufficient analgesia.** Insufficient analgesia was defined by an increase ≥1 on the Behavioral Pain Scale **(A)** or on the Richmond Agitation Sedation Scale **(B)**.

An insufficient analgesia level, defined by an increase >1 on the BPS, was recorded during 27 (68%) of the 40 endotracheal suctionings. Variations of physiologic data and behavioral scores related to the endotracheal suction are reported in Table 2. The variations of the pupil diameter during 10 mA, 20 mA and 40 mA stimulation were higher when analgesia was insufficient than when it was adequate using the BPS (Figure 3A). These results were confirmed when insufficient analgesia was observed using the RASS (Figure 3B).

**Table 2:** Variations of the physiologic data and behavioral scores induced by an endotracheal suction (n = 40) in 34 critically ill patients

	**Before endotracheal suction**	**After endotracheal suction**
Heart rate (bpm), mean (SD)	79 (11)	86 (10)
Mean arterial pressure (mmHg), mean (SD)	75 (5)	82 (7)
Respiratory rate (breath/min), mean (SD)	15 (1)	16 (1)
BPS, median (25-75^th^ percentile)	3 (3-3)	4 (3-5)
RASS, median (25-75^th^ percentile)	−5 (−5,-5)	−4 (−5, -4)

Comparison between before and after endotracheal suction (Wilcoxon rank sum test) was significant for all data (*P* <0.001). Bpm, beats per minute, *BPS* Behavioral Pain Scale, *RASS* Richmond Agitation Sedation Scale, *SD* standard deviation.

### Predictive values of tetanic stimulations for insufficient analgesia with BPS

**Figure 4:**
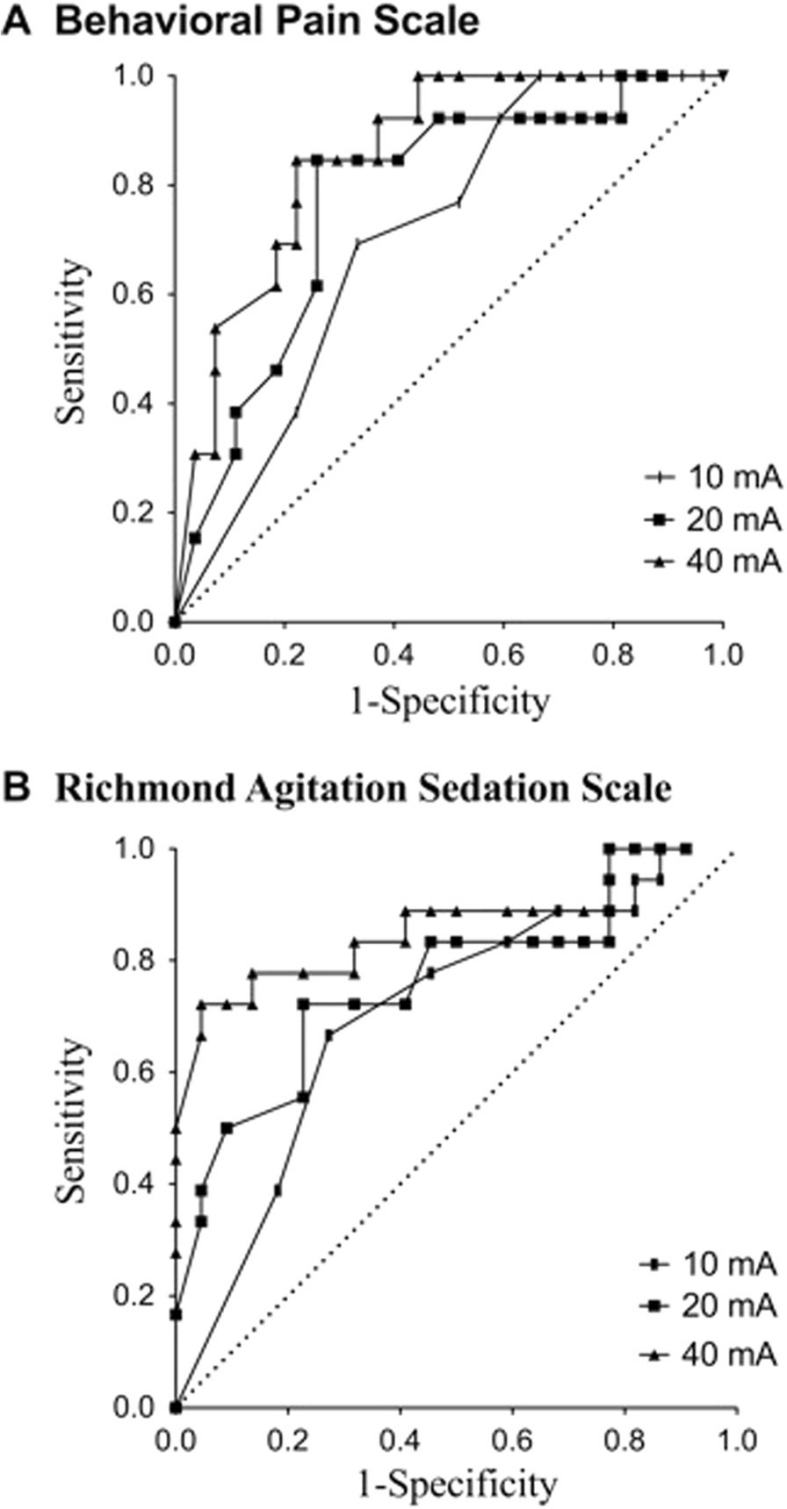
**Receiver operating curves for the prediction of an insufficient analgesia level before endotracheal suction by the measurement of the pupil diameter variations during 40 measurements in 34 critically ill patients after a 10, 20 or 40 mA tetanic stimulation.** Insufficient analgesia was defined by an increase ≥1 on the Behavioral Pain Scale **(A)** or in the Richmond Agitation Sedation Scale **(B)**.

Results are presented in Figure 4A and Table 3. The best cutoffs for the pupil diameter variation during 10, 20, 40 mA tetanic stimulations to predict an insufficient analgesia were 1, 5 and 13% respectively. The areas (95% confidence interval (CI)) under the receiver operating curve for predicting insufficient analgesia were 0.70 (0.54 to 0.85), 0.78 (0.61 to 0.91) and 0.85 (0.721 to 0.954) with 10, 20 and 40 mA tetanic stimulations respectively.

**Table 3:** Predictive values of pupil diameter variations for insufficient analgesia (BPS)

	**10 mA tetanic stimulation**	**20 mA tetanic stimulation**	**40 mA tetanic stimulation**
Best cutoffs for pupil diameter variation (%)	1	5	13
Sensitivity	0.69 (0.55 – 0.84)	0.85 (0.74 – 0.96)	0.85 (0.74 – 0.96)
Specificity	0.67 (0.52 – 0.81)	0.74 (0.61 – 0.88)	0.78 (0.65 – 0.91)
Positive predictive value	0.50 (0.35 – 0.66)	0.61 (0.46 – 0.76)	0.65 (0.50 – 0.80)
Negative predictive value	0.82 (0.70 – 0.94)	0.91 (0.82 – 1.00)	0.91 (0.83 – 1.00)
Youden index	0.36	0.59	0.62

All data except Youden index were 95% confidence interval. *BPS* Behavioral Pain Scale.

### Sensitivity analysis using RASS

Results are presented in Figure 4B and Table 4. The areas (95% CI) under the receiver operating curve were 0.70 (0.53 to 0.85), 0.76 (0.60 to 0.91) and 0.85 (0.69 to 0.97) with 10, 20 and 40 mA tetanic stimulations respectively.

**Table 4:** Predictive values of pupil diameter variations for insufficient analgesia (RASS increase ≥1 point)

	**10 mA tetanic stimulation**	**20 mA tetanic stimulation**	**40 mA tetanic stimulation**
Best cutoff for pupil diameter variation (%)	1	5	13
Sensitivity	0.67 (0.52 – 0.81)	0.72 (0.58 – 0.86)	0.78 (0.65 – 0.91)
Specificity	0.73 (0.59 – 0.87)	0.77 (0.64 – 0.90)	0.86 (0.76 – 0.97)
Positive predictive value	0.67 (0.52 – 0.81)	0.72 (0.58 – 0.86)	0.82 (0.71 – 0.94)
Negative predictive value	0.73 (0.59 – 0.87)	0.77 (0.64 – 0.90)	0.83 (0.71 – 0.94)
Youden index	0.39	0.49	0.68

All data except Youden index were 95% confidence interval. *RASS* Richmond Agitation Sedation Scale.

### Safety

**Figure 5:**
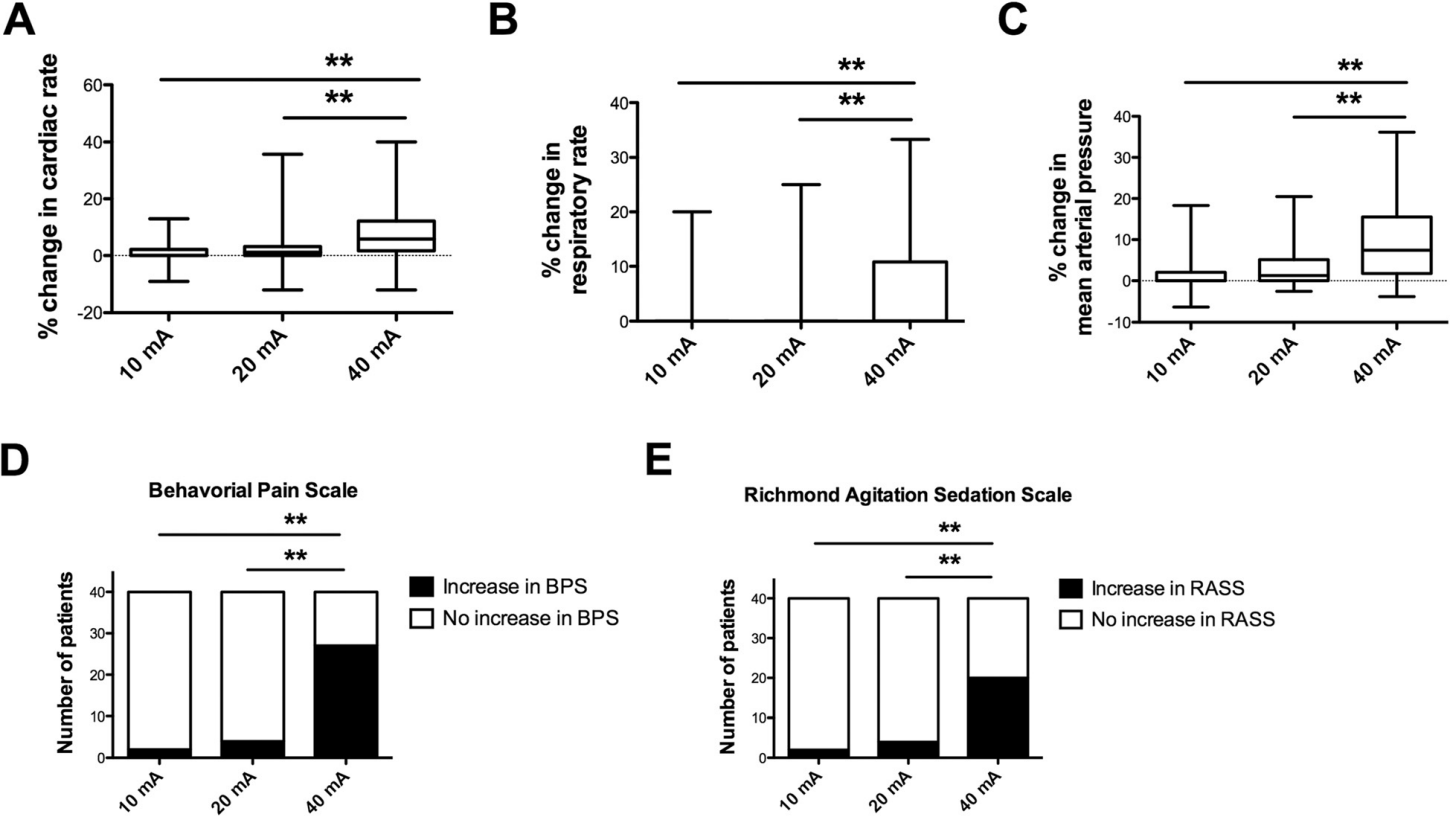
**Cardiac rate (A), respiratory rate (B), mean arterial pressure (C), number of patients with insufficient analgesia (D, E) after 10, 20 or 40 mA tetanic stimulation in 34 critically ill patients.** Insufficient analgesia was defined by an increase ≥1 point on the Behavioral Pain Scale **(D)** or on the Richmond Agitation Sedation Scale **(E)**.

A 10 or 20 mA tetanic stimulation were well tolerated without any significant variation of the physiological data (Figure 5A-C) and behavioral scales (Figure 5D-E).

Within the 40 procedures, significantly more patients experienced pain after 40 mA than after 10 or 20 mA stimulation (Figure 5D), these results were confirmed with the RASS (Figure 5E).

## Discussion

This study suggests that, in deeply sedated mechanically ventilated patients, quality of analgesia before procedural pain can be predicted by PDR measurement. A pupil diameter variation of 5% or more after a 20 mA tetanic stimulation was predictive of an insufficient analgesia before an endotracheal suction.

Most studies report that pain is rarely assessed in mechanically ventilated ICU patients [5,6,15]. The lack of pain assessment in ICU patients can lead to under- or overdosing of analgesics and hypnotics affecting patient outcomes. Analgesic underdosing leaves the patient with acute pain. This may alter the autonomic nervous system and therefore the myocardial function as well as adaptation to the mechanical ventilation. Pain-induced ventilator asynchrony may alter the outcome in the setting of traumatic brain injury, and acute respiratory distress syndrome [16]. Gelinas *et al*. reported that noxious procedures are frequently associated with asynchronous breathing [17]. Patient-ventilator asynchrony can confound attempts to deliver a lung-protective strategy or correct gas exchanges. It is also associated with adverse effects, including higher/wasted work of breathing, patient discomfort, increased need for sedation and confusion during the weaning process, prolonged mechanical ventilation, longer stay, and possibly higher mortality [16]. In addition, unrelieved pain induces prolonged stress on biologic systems and may predispose patients to adrenal insufficiency, alterations of immune function as well as glucose metabolism by increasing resistance to insulin. Pain may delay wound healing and ultimately it may lead to chronic pain [5,18,19]. On the other hand, analgesic and hypnotic overdosing is associated with consequences as severe as those of underdosing. Overdosing can lead to prolonged mechanical ventilation, prolonged ICU stay, opioids side effects (that is, ileus or nausea and vomiting) and withdrawal syndrome during drug weaning [20]. When transposing perioperative literature on opioid-induced hyperalgesia leading to chronic pain after surgery [21], it can be hypothesized that opioid overdosing in ICU can also lead to persistent pain after patient’s discharge from ICU.

Pain is rarely assessed, probably because it is difficult to evaluate the quality of analgesia in mechanically ventilated ICU patients who are unable to report their pain. It is challenging when patients are at rest but also before painful procedures [22]. The BPS scale is a validated useful score to evaluate pain in sedated ICU patients [11]. The present results corroborate those of Payen *et al*. [11] in confirming that BPS cannot predict the quality of analgesia before a painful procedure like endotracheal suctioning. Indeed, we found that the BPS score increased from 3 (3 to 3) at rest up to 4 (3 to 5) after endotracheal suction (*P* <0.001). In the study of Payen *et al*. [11], the BPS score significantly increased from 3.2 (3.1 to 3.3) to 4.9 (4.6 to 5.2) after endotracheal suction. We validated our results by using the RASS scale. We used this sedation scale considering agitation as a surrogate marker of pain because, in sedated patients, the evaluation of pain is challenging and increased agitation is usually the first clinical sign of pain. Physiologic responses (heart rate, arterial pressure, respiratory rate) could be other helpful tools to evaluate physiologic reactivity during noxious procedures in sedated critically ill patient, but the sensitivity of these clinical signs is poor [23] and cannot be used as a predicting tool.

PDR has been studied for nearly two decades as a potential marker of response to noxious stimulation in volunteers and surgical patients [8,17,23-28]. By incorporating a tetanic stimulator with the pupillometer, we were able to accurately record a 10 second time course of pupil diameter changes in response to a standardized painful stimulus. PDR has been mostly studied on anesthetized patients and had been shown to be a useful tool to predict the level of analgesia [9]. PDR had also been used to check the level of an epidural analgesia during general anesthesia [29]. As the mechanisms for PDR involve a sympathetic component, PDR might be different in anesthetized and unanesthetized conditions [30]. A very recent publication concluded that, regardless of the mechanisms, PDR in conscious patients following emergence from anesthesia was a reliable predictor of the level of analgesia [10]. As the mechanisms for PDR involve a sympathetic component, PDR might be different in anesthetized and unanesthetized conditions [30]. In ventilated patients, PDR is lower in heavily sedated patients (BIS <40) compared with lighter sedation (40 <BIS <60) [31]. The sedative regimen may therefore alter the PDR. In an exploratory subgroup analysis, the PDR during a 20 mA stimulation was not different in patients receiving propofol/sufentanyl compared with patients receiving midazolam/fentanyl (data not shown). Patients in ICU are sedated, which means that they are neither deeply anesthetized as for a surgical purpose nor fully awake. To the best of our knowledge, our study is the first to show that PDR can predict the quality of analgesia before procedural pain in deeply sedated patients. As in prior studies [9,23], we confirmed that pupil size is highly reactive to nociception and is a more sensitive tool when compared with other physiologic responses.

Several limitations of our study should be addressed. We measured the pupil diameter variation and not the absolute values of pupil size mostly because previous studies used the diameter change. Another reason is that the pupil size can vary with many conditions (drugs, light and so on). Deep sedation used in our study may have contributed to small changes in pupil size. In order to avoid potential interactions with ambient light, the pupillometer includes a silicone membrane surrounding the orbit, and the lighting in the room was controlled during the procedure in order to avoid light shining directly into the patient’s eyes. Some specific drugs may alter PDR measurement [24-27,32,33]. Droperidol and metoclopramide contract the pupil and reduce the PDR induced by the noxious stimulation. Larson recommends that when the PDR is used for monitoring the effect of opioids, antiemetic drugs acting on the D2 receptor should be avoided [26]. Clonidine also modifies central noradrenergic functions [25]. The patients included in the current study were not exposed to drugs interacting with the PDR. Traumatic brain injury patients could alter our conclusions; however, our primary endpoint was controlled by the use of two separate scales producing the same results, and our results could be considered important in this population in which controlling intracranial pressure is involved in the prognosis. Finally, there is a physiologic oscillation of PDR (10%) in the absence of any noxious stimulus, called pupillary hippus. The pupillary variations induced by the physiologic hippus are about 10% in awake patients with no pain. Even if the physiologic hippus may not be important in sedated patients, we cannot exclude that this phenomenon may interfere with our results.

## Conclusions

In conclusion, the quality of analgesia before procedural pain can be predicted by PDR measurement in deeply sedated mechanically ventilated patients. In this context, a pupil diameter variation value above 5% after a 20 mA stimulation has a high probability to be associated with insufficient analgesia during endotracheal suction. A 40 mA stimulation is poorly tolerated and should not be used in the setting of critically ill patients. Further studies are needed to validate the video pupillometer as a tool to guide analgesic administration in deeply sedated mechanically ventilated patients.

## Key messages

•A total of 68% of deeply sedated critically ill patients have pain-associated reactions during endotracheal suctioning, suggesting that a bolus of analgesic should be administrated before endotracheal suctioning.

•A pupil diameter variation value above 5% during a 20 mA stimulation in deeply sedated critically ill patients has a high probability of being associated with insufficient analgesia during endotracheal suctioning.

•While a 20 mA tetanic stimulation is well tolerated, a 40 mA tetanic stimulation is poorly tolerated and should not be used in the setting of critically ill patients.

## Abbreviations

BIS: Bispectral index; BPS: Behavioral pain scale; 95% CI: Confidence interval; ICU: Intensive care unit; mA: Milliamps; PDR: Papillary dilatation reflex; RASS: Richmond agitation sedation scale; SD: Standard deviation.

## Competing interests

The authors declare that they have no competing interests.

## Authors’ contributions

All the authors participated in the study management, data collection, and interpretation of data. JP, AR, KA and CL were responsible for the conception and design of the study, data interpretation and writing the manuscript. JT was responsible for data collection and data interpretation. HB was responsible for the interpretation and writing of the report. All authors had full access to all the data in the study, participated in the revision of the manuscript and all agreed to submit it for publication. All authors read and approved the final manuscript.
